# Brugmansia suaveolens Abuse in a Patient With Multiple Substance Use Disorders: A Case Report

**DOI:** 10.7759/cureus.106535

**Published:** 2026-04-06

**Authors:** Ilknur Gundogdu, Neriman Aras

**Affiliations:** 1 Department of Psychiatry, Samsun Mental and Nervous Diseases Hospital, Samsun, TUR

**Keywords:** abuse, anticholinergic, anticholinergic alkaloids, plant, substance use disorder

## Abstract

Anticholinergic pharmacological agents are commonly misused because of their euphoric effects, increased energy, improved mood, and enhanced social interactions. Some herbal products with anticholinergic properties can also be abused for their psychoactive effects. One such ornamental plant is Brugmansia suaveolens, which contains alkaloids including atropine and scopolamine and can produce toxic effects. This case report describes a male patient with Brugmansia suaveolens abuse and coexisting multiple substance use disorders. The patient reported consuming a decoction of Brugmansia suaveolens leaves and flowers as a daily tea for four years to obtain relief. He experienced anticholinergic symptoms, including drowsiness, sedation, agitation, ataxia, dry mouth, palpitations, fever, and visual and auditory hallucinations while under the influence of the plant. The patient was discharged following a four-week treatment course. Plants like Brugmansia suaveolens, which have anticholinergic but toxic properties, may be misused, especially by individuals with a history of multiple substance use disorders.

## Introduction

Acetylcholine plays a key role in parasympathetic regulation and is involved in various physiological functions, including motor activity, glandular secretion, cognition, and autonomic balance. At elevated doses, these agents can produce central and peripheral toxic effects, such as cognitive disturbances, perceptual abnormalities, autonomic dysregulation, and, in severe cases, potentially life-threatening complications. Although less commonly highlighted, anticholinergic agents have been reported among substances that are frequently misused, following major drug classes such as opioids, cocaine, cannabis, and amphetamines. Previous studies on anticholinergic misuse have reported prevalence rates of up to 34 percent; however, as noted in a recent review, a definitive prevalence is difficult to establish due to the growing number and variety of these agents and the fact that existing literature consists primarily of case reports. They are often favored because of their ability to induce euphoria, enhance energy and mood, and increase social interaction. Anticholinergic drug abusers are typically young, single, poorly educated, unemployed, have a history of substance abuse, and may have personality disorders.

Brugmansia suaveolens is an ornamental plant widely grown for its large, pendulous, trumpet-shaped flowers, which are typically white, yellow, or pale orange and often highly fragrant. Despite its attractive appearance, the plant contains potent tropane alkaloids, including atropine and scopolamine, which are responsible for its anticholinergic and toxic effects. Exposure to these compounds can produce a range of clinical manifestations, from peripheral symptoms such as dry mouth, tachycardia, and mydriasis to central effects, including delirium, hallucinations, and altered consciousness. This case report presents a patient with multiple substance use disorders who was abusing Brugmansia suaveolens, a plant with anticholinergic properties.

The patient gave consent for the publication of this case report.

## Case presentation

A 36-year-old male patient with multiple substance use disorders presented to an addiction clinic for treatment. He had begun using various substances, including cocaine, heroin, ecstasy, and THC, at the age of 16 and had previously received treatment at an addiction clinic on at least 10 occasions. His current medications included bupropion at a dose of 600 mg per day, which exceeds the recommended dose, along with a combination of buprenorphine/naloxone 8/2 mg per day. He continued to use THC once a month but had stopped using the other substances about six months prior.

The patient also reported using the leaves and flowers of Brugmansia suaveolens for four years, either by boiling them in water and drinking the infusion or by chewing them, one to five times per day, to achieve a high. He had discovered the plant’s effects after reading about it in a botanical book and requested that this information remain confidential. His last use of the plant was the day before hospitalization. He noted that the flowers produced stronger effects than the leaves. While under the influence, he experienced anticholinergic symptoms, including drowsiness, sedation, agitation, ataxia, dry mouth, palpitations, fever, and visual and auditory hallucinations.

There were no abnormalities observed in the physical examination, vital signs, or laboratory tests. During the psychiatric assessment, his speech was slow and disorganized, following a question-and-answer pattern, and he required more time to respond. His mood was depressive, and his thoughts were scattered. He exhibited persecutory and mystical delusions, as well as visual hallucinations. The patient was diagnosed with multiple substance use disorders and substance-induced psychosis, based on criteria from the Diagnostic and Statistical Manual of Mental Disorders, Fifth Edition (DSM-5).

Bupropion was discontinued due to psychotic symptoms and concerns about potential abuse. He was started on olanzapine (10 mg/day) and venlafaxine (75 mg/day), while the buprenorphine/naloxone combination was gradually tapered and then stopped. One week after hospitalization, he developed a hypomanic episode characterized by logorrhea, excessive speech, swearing, increased psychomotor activity, and insomnia. Consequently, venlafaxine was discontinued, and carbamazepine (400 mg/day), along with long-acting paliperidone palmitate injections (150 mg and 100 mg at one-week intervals), were added to his treatment. The psychotic symptoms and hypomanic state improved markedly after two weeks. The patient recovered and was discharged after six weeks, with no psychotic or mood symptoms observed during the first month post-discharge.

## Discussion

Individuals who abuse multiple drugs often use anticholinergic medications to enhance the effects of psychoactive substances such as alcohol, cocaine, benzodiazepines, and opioids. The prevalence of anticholinergic abuse in the general population remains uncertain. Research on anticholinergic drug abuse has primarily focused on psychiatric patients, with reported rates ranging from 6.5% to 34%. Anticholinergic drugs act as potent indirect dopamine agonists in the limbic system, an interconnected region including the nucleus accumbens and ventral tegmental area, which are involved in motivation, learning, memory, and reward processes. This mechanism may partly explain the abuse potential of anticholinergic drugs. They can alleviate depressive symptoms, enhance social skills, induce euphoria and relaxation, and boost energy levels by increasing dopaminergic activity. Additionally, their effects on muscarinic receptors in the central nervous system can produce visual and auditory hallucinations, delusions, euphoria, and even delirium.

A variety of natural psychoactive substances, including plants and fungi containing diverse alkaloids, have been identified. These substances can produce relaxing, stimulating, or hallucinogenic effects and have become an increasing concern in recent years. Brugmansia suaveolens, commonly called 'angel’s trumpet', is a small tree with showy, yellow, trumpet-shaped, fragrant flowers. It is widely cultivated as an ornamental plant for its tubular flowers in various colors and is found worldwide. Different parts of the Brugmansia plant are rich in tropane alkaloids, particularly atropine and scopolamine, which are also responsible for its toxic effects. The concentration of atropine and scopolamine in the nectar of the flowers is approximately 200 times higher than in the leaves. Brugmansia species have been used as hallucinogens and medicines in traditional practices since ancient times. The plant exhibits various therapeutic effects, including anti-inflammatory, cytotoxic, antioxidant, antibacterial, antispasmodic, antiasthmatic, anti-addictive, narcotic, and antinociceptive properties.

Poisoning from Brugmansia species is most often caused by accidental or excessive use. Clinical symptoms of poisoning include dry red skin, dry mouth, dilated pupils, headaches, irregular heartbeat, fever, convulsions, coma, urinary retention, acute mental changes, agitation, and hallucinations. There are two case reports of Brugmansia abuse in the literature. Göpel et al. reported three adolescents who were regular cannabinoid users and also abused Brugmansia. Visual hallucinations were observed in all three cases, similar to our case, but agitation and aggression were also present, unlike in our case. Gonçalves et al. described a case of schizophrenia involving the misuse of anticholinergic plants, including Brugmansia. Similar to our case, the patient, who had pronounced depressive symptoms, used the seeds of the plant rather than its flowers and leaves.

This is the third reported case of Brugmansia abuse to the best of current knowledge. It highlights a rare and unexpected form of substance abuse among patients with substance use disorders. Differential diagnoses include primary psychotic disorders, stimulant-induced psychosis, and delirium secondary to other toxic exposures. Plants containing anticholinergic substances, such as Brugmansia, can be misused by individuals with substance use disorders. In cases of suspected intoxication with unusual clinical features, the possibility of plant abuse should also be considered.

## Conclusions

Based on the available literature, only two similar cases have been reported, making this the third documented instance of Brugmansia misuse. As seen in this patient, long-term repeated use over several years can obscure the clinical picture by mimicking or worsening primary psychiatric symptoms, complicating both diagnosis and management. Thorough questioning about nonconventional substances, including plant-derived agents, may help improve diagnostic accuracy in such cases.
